# Applications of time-series analysis to mood fluctuations in bipolar disorder to promote treatment innovation: a case series

**DOI:** 10.1038/tp.2015.207

**Published:** 2016-01-26

**Authors:** E A Holmes, M B Bonsall, S A Hales, H Mitchell, F Renner, S E Blackwell, P Watson, G M Goodwin, M Di Simplicio

**Affiliations:** 1 Medical Research Council Cognition and Brain Sciences Unit, Cambridge, UK; 2 Department for Clinical Neuroscience, Karolinska Institutet, Stockholm, Sweden; 3 University Department of Psychiatry, Warneford Hospital, University of Oxford, Oxford, UK; 4 Mathematical Ecology Research Group, Department of Zoology, University of Oxford, Oxford, UK; 5 St Peter's College, University of Oxford, Oxford, UK

## Abstract

Treatment innovation for bipolar disorder has been hampered by a lack of techniques to capture a hallmark symptom: ongoing mood instability. Mood swings persist during remission from acute mood episodes and impair daily functioning. The last significant treatment advance remains Lithium (in the 1970s), which aids only the minority of patients. There is no accepted way to establish proof of concept for a new mood-stabilizing treatment. We suggest that combining insights from mood measurement with applied mathematics may provide a step change: repeated daily mood measurement (depression) over a short time frame (1 month) can create individual bipolar mood instability profiles. A time-series approach allows comparison of mood instability pre- and post-treatment. We test a new imagery-focused cognitive therapy treatment approach (MAPP; Mood Action Psychology Programme) targeting a driver of mood instability, and apply these measurement methods in a non-concurrent multiple baseline design case series of 14 patients with bipolar disorder. Weekly mood monitoring and treatment target data improved for the whole sample combined. Time-series analyses of daily mood data, sampled remotely (mobile phone/Internet) for 28 days pre- and post-treatment, demonstrated improvements in individuals' mood stability for 11 of 14 patients. Thus the findings offer preliminary support for a new imagery-focused treatment approach. They also indicate a step in treatment innovation without the requirement for trials in illness episodes or relapse prevention. Importantly, daily measurement offers a description of mood instability at the individual patient level in a clinically meaningful time frame. This costly, chronic and disabling mental illness demands innovation in both treatment approaches (whether pharmacological or psychological) and measurement tool: this work indicates that daily measurements can be used to detect improvement in individual mood stability for treatment innovation (MAPP).

## Introduction

For any disease or disorder, an essential part of treatment development is the ability to measure and assess key clinical outcomes. In the absence of appropriate techniques, innovation will be slow. One example of where this problem has hampered treatment development is bipolar disorder. Bringing together ideas from several areas of science—here psychology, psychiatry and applied mathematics—may provide an opportunity for treatment advances.

Bipolar disorder (formerly ‘manic depression') is characterized by repeated episodes of depression with at least one (hypo)manic episode of elevated mood and overactivity.^1^ The clinical picture is that depression tends to dominate; therefore, depressed mood fluctuations present the focus of this paper. Co-morbid anxiety is common, fuelling depression, and relates to poorer prognosis.^2^ About 1% of adults have a lifetime history of bipolar I or II disorder,^3^ which carries the highest rate of suicide of all psychiatric disorders.^4^ The primary treatment is Lithium,^5^ a pharmacological treatment established over 40 years ago,^6^ and satisfactory only for a minority of patients.^7, 8^ Despite research, treatment advancement has been slow and a substantial proportion of patients remain highly symptomatic. The development of new research methods to support treatment innovation is critical.^9, 10^

Treatment innovation for bipolar disorder has been hampered by the lack of techniques to capture adequately one of its key clinical features—ongoing mood swings (referred to as mood instability henceforth) that can persist at a subsyndromal level. There are several problems associated with traditional approaches to bipolar disorder: an exclusive focus on full-blown illness episodes; infrequent measurement; retrospective reporting biases; and a lack of mathematical tools to capture mood over time. Treatment advances with medication have been made on the basis of studies of acute episodes (of depression or mania) and longer term relapse prevention studies. The necessary programme of clinical research is daunting and has rarely been attempted, except, for example, by extending an existing marketing authorization for schizophrenia, which offers a simpler development path. By analogy, we contrast the current clinical picture in bipolar disorder with advances for another chronic condition such as diabetes, where ongoing monitoring of fluctuations in glucose levels helps to prevent full-blown illness episodes. As yet, bipolar disorder lacks the equivalent of more frequent glucose profile monitoring.

Traditionally, outcome in bipolar disorder has focused on infrequent measurements such as counting full-blown mood episodes, which occur on average less than once per year.^11^ Such a sampling rate takes a long time in the life of an individual patient who would prefer to know in much shorter time frames (days/weeks rather than months/years) how their illness is progressing. Further, infrequent mood assessments over long time periods yields impoverished/noisy data. This problem has bedeviled treatment trials for bipolar disorder, and as a result some have had to be terminated early.^12^ Infrequent outcome measurement bears the risk of failing to capture the real impact of interventions and has driven recent efforts to develop more ecologically valid outcome measures in pragmatic effectiveness trials, such as frequency of clinical adjustments in medication.^13^ Assumptions about mental health treatment may be contributing to the field being stuck. Simply taking a small number of measurements pre- and post-treatment has worked well for other mental health disorders (such as anxiety disorders) where symptoms fluctuate less and a simple reduction in scores equates with treatment success. However, bipolar disorder is different as symptom reduction can be temporary and reverse (for example, the next week or the next day), and a large reduction can even herald greater instability to follow. Thus, more traditional measurement approaches (for example, unitary measurements pre- and post-treatment) commonly used in other psychiatric disorders neglect bipolar patients' difficulties with ongoing mood instability.

A new approach for bipolar disorder is to focus on the chronic subsyndromal ‘mood instability' that persists in between full-blown mood episodes,^14, 15^ impairing daily functioning^16, 17^ and worsening long-term prognosis.^18^ Recent initiatives have measured mood weekly,^19^ but, various limitations have emerged. If the frequency of mood swings is different from the sampling rate, information can be missed (‘sparse sampling').^20^ Memory is mood-state-dependent,^21^ thus having to remember symptoms from the past 7 days introduces a retrospective reporting memory bias driven by current (fluctuating) mood state,^21^ particularly in a disorder associated with memory deficits.^22, 23^ Further, the number of weekly data points needed to apply a time-series approach to capture mood instability requires patients to comply with monitoring for at least 6 months (with little missing data). This is hard for a group where compliance with a regular regime presents a core problem and long-term monitoring is onerous. At an individual patient level, patients require a more rapid answer to whether or not a new treatment is proving helpful, particularly so when medication side effects can be burdensome,^24^ and where unfortunately some antidepressant medications can precipitate symptom *worsening.*^25^

Here, we suggest a bipolar treatment outcome measure of ongoing mood instability using a *daily sampling regime* over 1 month in daily life, and further to analyse such mood data via an applied mathematical time-series approach. This alternative approach, that is, using mood data sampled daily to test for improvements in mood instability over time, is less prone to memory and mood biases than longer time intervals. It captures ongoing symptoms in the real world rather than only in the clinic. Daily sampling has proved effective as a self-monitoring tool.^26^ Here we adopt it for the first time as a method for improving treatment outcome assessment. Sampling for 1 month is a commonly used time frame in clinical practice (for example to reveal when drug side effects become obvious to patient and clinician)^27^ and could be incorporated in daily routines (via technology such as smartphones). Time-series approaches^28^ allow changes in mean mood and variability in mood to be properly characterized. This provides a powerful way to extract differences in mood profiles before and after treatment in patients with bipolar disorder by evaluating whether differences in the goodness of fit of models have different time-series structures.^28, 29^ It introduces mechanism into the understanding of mood variability at the level of cognition (mechacognition).^29^ Ultimately, this may promote more rapid examination of new early-stage treatments, or treatment changes for an individual patient, by targeting clinically meaningful ongoing and persistent subsyndromal symptoms.

Here we investigate the use of a daily mood-monitoring time-series approach in a single case series study of a novel treatment for bipolar disorder. Single case series designs are important research tools in the development of new treatments, providing an alternative to cohort and case–control designs.^30^ They require smaller sample sizes compared with randomized control trials, as statistical power is provided by the within-subject comparison of treatment effects.^31, 32, 33^ Moreover, single case series allow for further refinement of treatment protocols at an exploratory stage, when investment of time and resources required by randomized control trials would be premature. They also allow us to examine individual patient level data — our clinical goal.

The broader clinical picture informing our choice of treatment innovation concerns the high rates of anxiety that complicate bipolar disorder, flagged by clinical guidelines as an unmet need requiring treatment innovation.^34^ Anxiety symptoms are associated with worse prognostic factors such as rapid mood cycles, higher illness severity, less euthymic days and increased suicidality,^35^ poorer functioning and worse treatment response.^36^ Unfortunately, pharmacological treatment for anxiety within bipolar disorder has not been investigated formally and antidepressants may even destabilize mood, for example, inducing a ‘manic switch'.^37^ Psychological treatments such as cognitive therapy offer adjunctive approaches for addressing anxiety in bipolar disorder,^38^ but, like the use of antidepressants, often represent extrapolation from uncomplicated anxiety disorders.

Anxiety in bipolar disorder should be of particular interest if, as we have proposed, it contributes to depressed mood instability via an ‘emotional amplifier' effect of anxiety-laden mental imagery.^39^

Mental imagery occurs when ‘perceptual information is accessed from memory, giving rise to the experience of ‘seeing with the mind's eye', ‘hearing with the mind's ear' and so on'.^40^ Recent findings suggest that imagery can have an important role in the development and maintenance of various mental disorders, including anxiety disorders.^41^ Traditional clinical assessments can neglect asking patients about their intrusive upsetting mental images,^42^ such as intrusive images of dreaded future events or negative memories, images which can fuel anxiety and low mood. A psychological treatment targeting such maladaptive imagery could therefore improve anxiety and mood instability.^39^ Here we test one such protocol (MAPP; Mood Action Psychology Programme: clinicaltrials.gov identifier NCT01981018), which is an imagery-focused cognitive therapy.

MAPP consisted of three distinct phases: first, an in-depth four-session assessment was conducted in which a clinically significant imagery-related anxiety target formulated to impact on mood instability was jointly identified by the patient and co-therapists as a treatment target. For example, an intrusive image of a past stressful event, or a ‘flash-forward' image to an anxiety-provoking situation. Second, in the active treatment phase, imagery-based psychological techniques were applied to address this treatment target.^43^ Depending on the formulated target, aims of the treatment phase included (1) transforming or dampening problematic, destabilizing intrusive imagery via imagery rescripting or competing visuospatial tasks; (2) changing the patient's understanding of imagery, thereby reducing its impact via the use of metacognitive techniques (for example, having an image of an event does not mean it is real); (3) increasing access to positive, mood-enhancing or soothing imagery using positive imagery strategies (for example, compassionate self-view of looking after oneself in the future). The treatment focus was on learning a small number of techniques well. Techniques were tailored to break patients' bespoke symptoms cycles, with the aim of preventing future anxiety and mood extremes. Third and finally, a consolidation phase was designed to enhance learning and recall of the treatment, and included creation of a video ‘blueprint' to act as a record of the main learning outcomes for the patient to refer back to in the future and potentially share with others.

Using a non-concurrent multiple baseline design case series of 14 patients with bipolar disorder who undertook the new treatment, we hypothesized that this novel treatment (MAPP) would lead to the following:

- for the whole sample combined, reductions in mean levels of mood and anxiety (weekly and daily), as well as improved daily mood instability (that is, a more regular temporal structure) over the new treatment;
- an improvement at the individual patient level, whereby daily mood instability profiles would become more stable over treatment.

The study assessed adherence to daily mood monitoring. In addition, we examined whether mood improvement was mediated by changes in the psychological treatment target (imagery). A further outcome measure was relapses assessed by clinical interview at 24 weeks post-treatment versus pre-treatment (for additional clinical measures and associated results, see Supplementary Materials). Overall, there are two forms of innovation in the current study: an examination of a novel treatment (MAPP), and a new measurement tool for individual mood instability (daily mood monitoring).

## Materials and methods

### Experimental design

A non-concurrent multiple baseline design, with a series of A-B replications was used.^32, 44^ Fourteen patients with bipolar disorder were randomly assigned to 4, 5 or 6 weeks baseline (*n*=5, 4 and 5, respectively) using a blocked randomization procedure by an independent researcher. Weekly mood monitoring was completed via a web-based system throughout baseline and continued until 24 weeks post-treatment. Daily mood monitoring measures were completed via the web-based system for two time periods: the last four baseline weeks (pre-treatment),^45^ and the 4 weeks immediately after ending treatment (post-treatment). To allow assessment of the structure of the correlation in the time-series, participants were required to complete a minimum of 23/28 daily assessments at baseline for inclusion into the study (pre-specified). Pen-and-paper measures were completed at five face-to-face assessments: at pre-treatment, at the end of treatment, and at the 4, 12 and 24 weeks follow-ups. Thus, there were pre-defined end points and rules for stopping data collection. To reduce bias, an assessor other than the treatment therapists completed the outcome assessments.

Sample size was pre-specified as 15 cases (clinicaltrials.gov identifier NCT01981018), based on pilot results leading to an effect size estimate of *d*=0.8. Recruitment was via referral from local mental health services. Interested participants underwent a face-to-face eligibility assessment (*n*=28). Recruitment stopped once 15 met inclusion criteria. One person withdrew after two sessions due to severe side effects of Lithium precluding further involvement. The final sample comprised 14 patients (Table 1 and Supplementary Table S1). Further details of recruitment and screening are provided in the Supplementary Materials. Ethical approval was granted by NRES Committee East of England — Essex (13/EE/1074). Informed consent was obtained from all the participants after the nature and possible consequences of the study were explained.

### Research objectives

The study aimed to investigate the delivery and efficacy of a new cognitive therapy treatment for bipolar disorder (imagery-focused), studying patients one-by-one in a ‘case series' and assessing their mood over time. The primary outcome measures were pre-specified as change in weekly scores of anxiety, Beck Anxiety Inventory (BAI),^46^ and depression, Quick Inventory of Depressive Symptomatology Self-Report (QIDS-SR),^47^ over treatment: clinicaltrials.gov identifier NCT01981018. Pooled scores from aggregated time points over the 4 weeks after treatment (post-treatment) were compared with aggregated time points over the pre-treatment baseline (weekly QIDS-SR and BAI).

We sought to examine, for all participants combined, whether their mean aggregated daily mood and anxiety improved from pre- to post-treatment, and whether the temporal structure of mood scores improved — calculated as a time-series profile of daily mood on the QIDS-SR scale (based on earlier work over a broader time scale).^28^ Further, we aimed to analyse individual patient time-series mood profiles for improvements in mood instability and transition between mood states. We examined whether imagery treatment target ratings were associated with changes in the primary outcome measures.

To confirm that there was no significant reduction in symptoms pre-treatment, consistency of depression and anxiety was assessed over the baseline period. Adherence to daily monitoring was assessed for 28 days pre- and post-treatment.

Additional objectives were to assess intervention effects on clinical relapses of mania and depression, and anxiety co-morbidity, as well as self-reported affective lability, impairments in functioning, hopelessness and suicidality, and medication compliance (see Supplementary Materials).

### Measures

#### Weekly mood monitoring

Depressive symptom severity over the past 7 days was assessed using the QIDS-SR,^47^ a 16-item questionnaire covering the nine DSM IV-TR^48^ major depressive disorder symptoms. Ratings are made on a four-point scale (0–3) anchored at all points by a description. For example, Question 11, ‘view of myself' is anchored at 0=‘I see myself as equally worthwhile and deserving as other people', 1=‘I am more self-blaming than usual', 2=‘I largely believe that I cause problems for others' and 3=‘I think almost constantly about major and minor defects in myself'. Following local ethics committee advice, item 12 ‘thoughts of death or suicide' was removed from the online mood monitoring (weekly and daily). Note: the complete scale including suicide item was used for face-to-face assessments. The QIDS-SR correlates highly with established clinician rating scales such as the Hamilton Rating Scale of Depression (*r=*0.86) and has high internal consistency (*α*=0.87).^47^

Anxiety symptom severity over the past 7 days was measured using the BAI,^46^ comprising 21 items. Ratings are made on a four-point scale (0–3) from ‘not at all' to ‘severely'. Example items include ‘scared', ‘fear of losing control' and ‘heart pounding or racing'. The BAI has high internal consistency (*α*=0.92) and test–retest reliability over 1 week, *r*=0.75.^49^

#### Daily mood monitoring

Depressive symptoms were assessed using a modified version of the QIDS-SR^47^ anchored to the last 24 h. The suicide item was again removed, as were two items referring to an unfeasible time frame for a daily rating—‘decreased/increased weight within the last 14 days', yielding 13 items. The structure of the daily QIDS-SR scoring is appropriate for time-series analysis with Gamma errors (see Supplementary Materials and also ref. 28). For further individual patient analysis of the time-series (using Markov chains — see the methods below), the cut-off score for moderate depression on the original QIDS-SR (⩾11)^47^ was adapted to ⩾9 to account for the omitted items.

Daily anxiety symptoms were assessed via two bespoke ratings on a similar scale: the extent to which the participant felt anxious and fearful, and the severity of physical anxiety symptoms.

#### Computerized monitoring system

The computerized monitoring system was implemented via two secure servers: a web server hosting the website for completing questionnaires, and a database server to generate automatic prompts. Automated prompts were sent via email to all the participants (*N*=14), and by a mobile phone short message service where requested (*N*=12). If a participant did not complete a weekly or daily measure online within the scheduled time frame, researchers would attempt to contact them to complete the measure at the earliest opportunity, either via the online system, phone or at a therapy session. At pre- and post-treatment combined, 27 outstanding primary outcome weekly assessments (of 504, that is, 5.36%) and 34 outstanding daily assessments (of 784, that is, 4.34%) were collected following this procedure.

#### Additional measures

Measures taken at the face-to-face pre- and post-treatment assessments and weekly scores of mania symptoms are reported in the Supplementary Materials.

### Treatment

The imagery-focused treatment consisted of three phases: an in-depth assessment (four sessions—‘mapping'), active treatment and consolidation (in total 10–14 sessions, including mapping). During the fourth and final assessment session, the patient and co-therapists jointly agreed on a clinically significant target for imagery-focused cognitive therapy, for example, ‘destabilizing' imagery-related anxiety or lack of positive imagery. Choice of target was based on the following criteria: (1) the symptom was a valid target in its own right (that is, it was distressing or destabilizing), (2) it was formulated to have a plausible link to mood instability and (3) it was judged to be tractable in a brief imagery-focused cognitive therapy intervention. The active treatment phase (~4–6 sessions) selected imagery intervention strategies, either alone or in combination: metacognitive, imagery rescripting, positive imagery and competing tasks. Metacognitive strategies teach the patient to view images as merely mental representations (‘an image is just an image') rather than imbued with emotional meaning. Imagery rescripting helps the patient to transform distressing or maladaptive images into more benign, functional ones.^50^ Positive imagery strategies involve the creation and practice of mood-enhancing,^51^ or soothing images.^52^ Imagery competing strategies use concurrent visuospatial tasks (such as a computer game) to dampen problematic imagery.^53, 54^ In the consolidation of learning phase (two sessions), patients made a video record of important parts of their therapy. Two co-therapists were present in all the sessions (sometimes one remotely via Skype).

Participants attended a mean of 11.6 sessions of therapy including the assessment in the mapping phase, active treatment and consolidation (s.d.=1.22; range 10 to 14), with a mean duration of 15.6 weeks (s.d.=2.77; range=11 to 22).

### MAPP Manual

The treatment followed a manual developed by mental health clinicians with expertise in bipolar disorder and Cognitive Behavioural Therapy, with feedback from clinicians external to the project and patients with bipolar disorder.

### Statistical analysis

#### Data completion at face-to-face assessments

All the 14 participants took part in the face-to-face assessments at pre-treatment baseline and end of treatment, 4, 12 and 24 weeks post-treatment. At the end of treatment assessment, one participant did not complete the questionnaires (missing data).

#### Weekly mood-monitoring scores

The comparison of average weekly baseline scores (QIDS-SR and BAI) with those at follow-up were conducted using paired two-tailed *t*-tests, following checking of the distributions of difference scores. Pooled weekly scores from aggregated time points over the pre-treatment baseline were compared with aggregated time points over the four weekly scores post-treatment. Note pre-treatment baseline was randomized as 4, 5 or 6 weeks (see Experimental Design).

Missing values for the primary outcome weekly measures were low: 1 of 70 QIDS-SR assessments at baseline (completion rate 98.57%). At post-treatment, 2 of 56 QIDS-SR were missing (completion rate 96.43%). At baseline, 2 of 70 BAI assessments were missing (completion rate 97.14%). At post-treatment, 2 of 56 BAI were missing (completion rate 96.43%).

#### Daily mood monitoring

#### Daily mood and anxiety scores

The comparison of average daily baseline scores (QIDS-SR and anxiety ratings) with those at follow-up were conducted using paired *t*-tests (two-tailed), following checking of the distributions of difference scores. Each assessment questionnaire incorporates both scales. Missing values for the daily measures were low: at baseline, 17 of 392 assessments were missing (completion rate 95.66%). At post-treatment, 17 of 392 assessments were missing (completion rate 95.66%). Overall completion rate was 750 out of 784 (95.66%).

#### Daily mood profiles

For further details of the time-series analysis on daily mood profile described in the Results, see also Supplementary Materials.

#### Effect sizes

Effect size (Cohen's *d*) was calculated by dividing the mean difference by the pooled s.d.^55^ Effect sizes of nonparametric tests were calculated by computing *r*=z/SQRT(*n*) (ref. 56) and *r* was converted to *d* using the following formula: *d*=(2 × *r*)/SQRT(1−*r*^2^).^57^

#### Outliers

Analysis of outliers was incorporated into analysis of the normality of residuals obtained from the fitting of parametric *t*-tests and analysis of variance and the associated use of nonparametric tests, as required. No outliers were excluded. If there was skewness, they were downweighted using nonparametric tests.

#### Time-series

Correlation structures across the pre- and post-treatment time periods were analysed using time-series analysis following Bonsall *et al.*^28^ On the basis of a conditional-likelihood framework (see Supplementary Materials), we fitted linear and nonlinear (threshold based) autoregressive (AR) models to the mood scores before and after treatment. A linear autoregressive model with a single lag (referred to as AR(1) model) takes the form:


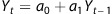


where *Y*_*t*_ is the mood score on the current day (at time *t*) and *Y*_*t*−1_ is the mood score from the previous day (at time *t*−1). *a*_0_ and *a*_1_ are unknown parameters (to be statistically estimated). A threshold autoregressive model (with a single lag—referred to as a threshold autoregressive structure (TAR(1)) model) takes the form:


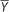


where *Y*_*t*_ is the mean of the mood score and the model has different parameters ([*a*_0_, *a*_1_] or [a_2_, a_3_]) above and below this mean mood score. The distribution of mood scores is non-normal and in the Supplementary Materials (and elsewhere^28^) we have shown that these scores are well-characterized by a Gamma distribution. We use this probability distribution to construct an appropriate conditional likelihood (see Supplementary Materials). Computationally, we minimize the negative log-likelihood using an expectation-maximization method to deal with missing values (outliers) within a modified simplex optimization algorithm implemented in C. Using the Akaike Information Criterion,^58^ we evaluate the goodness of fit of four different time-series models: AR(1), AR(2), TAR(1) and TAR(2) to the pooled participant pre- and post-treatment time-series. We also determine one-step-ahead predictions for each time-series based on the overall best fit pre- or post-treatment time-series model.

#### Markov chain analysis

Individual variation in QIDS-SR scores was analysed as a Markov chain stochastic process. We group QIDS-SR scores into three countable values (0, <9, ⩾9) and determine the transition probability from a QIDS-SR score on day *t* to a QIDS-SR score on day *t*+1. This gives a 3 × 3 one-step transition matrix:


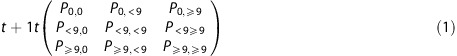


where each entry, *P*_x,y_, is a transition probability from one day to the next; for example, *P*_0,0_ is the probability that given a QIDS-SR score=0 on day *t*, the QIDS-SR score on day *t*+1 is also 0; and *P*_<9,0_ is the probability that given a QIDS-SR score <9 on day *t*, the QIDS-SR score on day *t*+1 is 0.

For each participant, we determine transition matrices for probability of changes in QIDS-SR scores pre- and post-treatment. Using standard methods of analysis,^59^ we solve these matrices for the stationary probabilities of QIDS-SR score states pre- and post-treatment.

### Code availability

Scripts used for the time-series and Markov chain analyses are available on request from the second author (MBB).

## Results

### Patient characteristics

The characteristics of the study cohort are shown in Table 1 and Supplementary Table S.1.

### Treatment intervention reduces weekly mood monitoring scores

Pooled weekly scores (depression/anxiety) from aggregated time points over the pre-treatment baseline (4/5/6 weeks) were compared with aggregated time points over the corresponding four weekly scores post-treatment (primary outcome measures: see Materials and methods; Experimental design).

Paired two-tailed *t*-tests confirmed that there was a significant reduction in both patients' mean depression, QIDS-SR,^47^ and anxiety scores, BAI,^46^ from pre- to post-treatment, see Table 2 (and Supplementary Figure S.1. for individual level data including 6-month follow-up).

During the pre-treatment baseline (alone), symptoms of depression (QIDS-SR) and anxiety (BAI) showed no indication of spontaneously improving over time (see Supplementary Results; weekly mood-monitoring scores remain consistent over the pre-treatment baseline periods). This indicates that the observed effects at post-treatment are more likely owing to the intervention rather than the passage of time.

### Daily mood monitoring adhered to for 28 days pre- and post-treatment

All the patients successfully completed pre-treatment baseline monitoring (that is, at least 23 out of 28 daily mood measures) in the 4-week active run-in phase of the study, indicating excellent adherence (*M*=26.79, s.d.=1.42). Likewise, all patients successfully completed post-treatment daily mood monitoring (*M*=26.79, s.d.=1.97).

### Treatment intervention reduces mean daily mood-monitoring scores

Paired two-tailed *t*-tests showed significant reductions in mean daily QIDS-SR and anxiety scores in the 28 days pre-treatment versus post-treatment, see Table 3.

Henceforth, we focus on daily QIDS-SR for our analysis of mood instability; see Materials and methods for psychometric properties relevant to the time-series.

### Treatment intervention affects temporal structure of daily mood profiles: aggregated patient analysis

**Figure 1:**
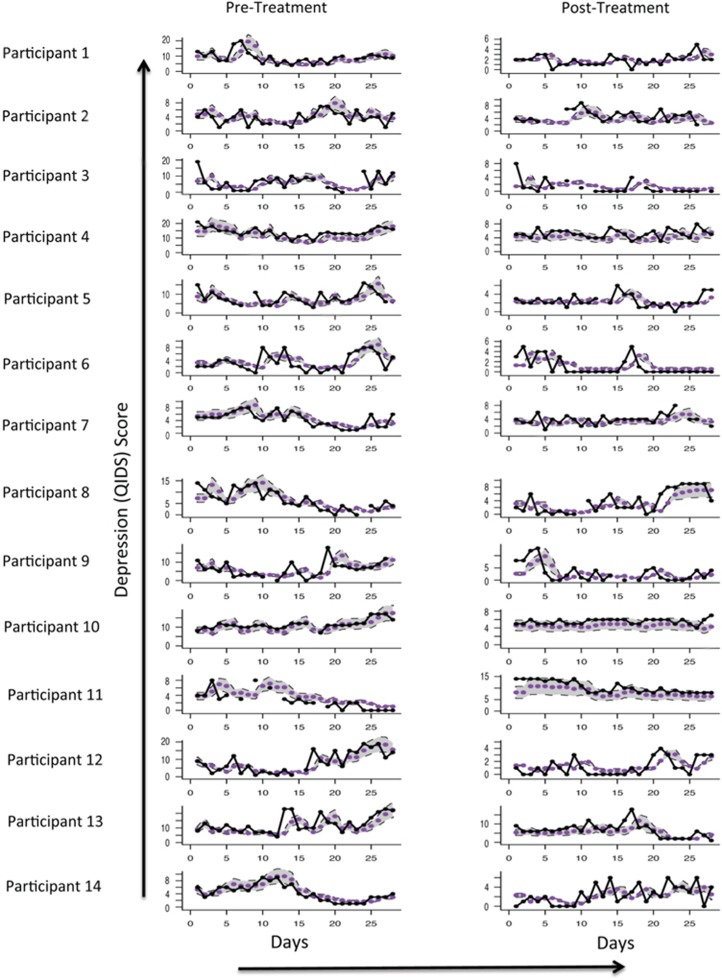
The QIDS-SR daily mood scores for 28 days pre-treatment (left hand side) and 28 days post-treatment (right hand side), per participant. Participants presented in order of starting mood monitoring. Individual mood plots show the QIDS-SR score (black points and black line), best model fit from time-series analysis (purple points). Predicted values (from the overall time-series model pre- and post-treatment) are shown with an approximate 95% CI band in grey. Note, differing y axis are used for visibility of any change in variability of the daily ratings (and see Supplementary Figure S1 for mean weekly values pre- and post-treatment). CI, confidence interval; QIDS-SR, Quick Inventory of Depressive Symptomatology.

Aggregate mood profiles before and after treatment showed different temporal dynamics. Our time-series analysis through appropriate model selection using Akaike Information Criterion (see, Supplementary Figure S.2.) revealed that before treatment the aggregate mood profiles were most appropriately described with a TAR model. TAR models represent a class of time-series models that characterize dynamics that have complex correlation structures and as such are likely to predict highly nonlinear changes in mood scores. Across the 14 patients, the TAR model predicts different mood dynamics above and below a threshold (in our case the mean) QIDS-SR score and gives rise to daily mood profiles that are highly variable (Figure 1). Pre-treatment, the patient time-series were appropriately described with four parameters based on two previously reported daily mood scores to predict current mood score (see Supplementary Table S.2.).

Post-treatment, the aggregate mood profiles were approximated with a simpler, less complex, time-series model that only required two parameters based on two previously reported daily mood scores to predict current mood score (see Supplementary Table S.2.). That is, the mood dynamics no longer differed above and below a mean QIDS-SR score.

### Treatment intervention affects individual daily mood-instability profile

Time-series models derived from aggregated scores of all 14 patients were fitted to individual patients. Comparisons of these individual mood profiles before and after treatment (together with goodness of fit criteria) for the pre-treatment model (TAR(2)) and the post-treatment model (AR(2)) are shown in Figure 1.

**Figure 2:**
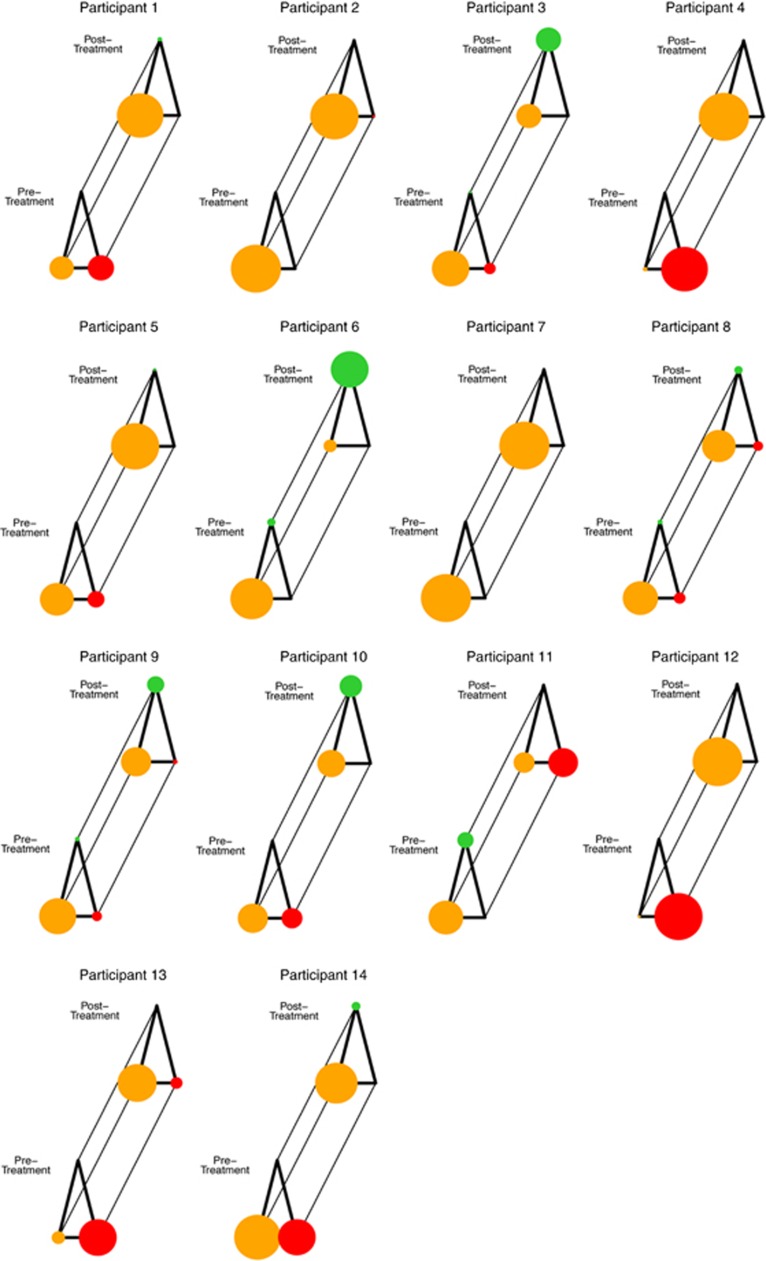
Markov chain analysis of changes in QIDS-SR daily scores for individual participants pre- and post-treatment. Circle size represents the probability of a patient being in a certain mood state: red circles represent moderate levels of depression (QIDS-SR⩾9); orange circles represent mild levels of depression (QIDS-SR⩽9 and not equal to 0); green circles represent the absence of any depressive symptoms (QIDS-SR=0). For a given participant, this gives a picture of transition between states during their 28-day baseline phase (front triangle) which can be compared with their 28-day post-treatment phase (back triangle).

Mood profiles before and after treatment showed changes in the overall instability pattern (see Supplementary Table S.3.). To investigate how changes in the temporal structure affected the mood profile, the probability of moving between different mood states, we determined the probability transitions between three QIDS-SR score states (zero, mild and moderate levels), (see Materials and methods; Daily mood monitoring). At an individual patient level, our analysis revealed a general increase in the probability of observing patients with low QIDS-SR scores (<9) after treatment and a reduction in the probability of high QIDS-SR scores (⩾9). Individual patient analyses were possible given the appropriate monitoring before and after treatment, and the details of this approach (using a Markov chain analysis) and the results for each patient are reported in Figure 2 (and individual level data in Supplementary Table S.3.). Figure 2 shows the general patterns of changes in the long-term probabilities of QIDS-SR scores in each of the three states (zero, mild and moderate levels) before and after treatment per patient. This analysis showed that in 11 of 14 cases, there was an improvement in mood profiles with a decreased occurrence of high QIDS-SR scores (>9), and hence less time spent above the clinical cut-off for depressed mood. Further, 7 out of 14 patients had more zero QIDS-SR scores post-treatment, and hence had no reported mood dysfunction (see Figure 2).

## Discussion

Bipolar disorder, a severe and chronic illness, urgently needs the rapid development of novel treatments and measurement innovations. Subsyndromal mood instability is a key clinical factor impacting on the long-term course of bipolar disorder; however, it has remained a neglected treatment target, and techniques to measure mood instability are lacking. Our study showed that time-series analysis of mood scores collected daily for 1 month can capture mood instability, and its improvement, in a case series of patients with bipolar disorder. The clinical application of mathematical approaches may open solutions to a critical clinical challenge, highlighting the benefit of translating advances from other disciplines to treatment innovation in mental health.^10^

Our study cohort showed excellent adherence to the daily mood sampling regime both pre- and post-treatment, suggesting that this is a viable strategy in bipolar disorder. Monitoring in daily life may help avoid the memory biases inherent when reporting over longer time intervals (for example, weekly or at clinic appointments). One participant stated ‘[I] liked the daily one, [but] found the weekly one unhelpful as it is very difficult to remember a whole week' another participant stated ‘psychiatrists always ask when is the last time you felt like this – but they don't give you the tools to answer it, it's all based on memory and memory is the least reliable aspect here…when you are sick the memory itself is distorted'. A longitudinal time-series approach, as developed here, may help in part to redress these sorts of patient concerns.

Time-series models enabled us to obtain a reliable representation of subsyndromal mood instability aggregated over the whole group, and at an individual patient level. At the aggregated level across the whole group, we show that time-series differences in mood score occur before and after treatment. Clinically, before treatment, patients had complex correlated mood scores (described statistically with a threshold autoregressive TAR(2) model). After treatment, patients had simpler, less variable mood dynamics (described by autoregressive (AR(2)) model). As such and potentially clinically, for an individual patient (and those around them) post-treatment, it may be sufficient to predict mood dynamics with a few symptoms and/or based on data collected over a smaller number of days than compared with pre-treatment conditions (see Figure 1).

At the individual patient level, Markov chain analysis provided descriptions of treatment effects in terms of time spent in a given mood state, and likelihood of transitions between mood states. The associated figures for each patient provide a simple demonstration of the probabilistic transition between the three types of mood states (absent/mild/moderate depressive symptoms, see Figure 2). Providing patients with such representations could help better understanding of their own mood symptoms (a critical demand of bipolar patients) and reinforce treatment adherence, a major obstacle in this population. Such tools could allow symptom improvement/deterioration to be checked by clinician or the patient themselves.

The case series suggests that the novel therapy (MAPP: imagery-focused cognitive therapy) may be a promising treatment for further development, reducing mood instability for 11/14 patients, and improving mood and anxiety symptoms at a group level. The anxiety reduction is encouraging as this is an unmet need in bipolar disorder, supporting the idea that reducing anxiety may provide a route to reducing mood instability. The high attendance rate speaks about the intervention's acceptability. The improvement in mood instability shown by the less complex temporal structures of mood profiles post-treatment is reinforced by convergent improvements on more traditional measures (Supplementary Table S.4.). Secondary outcomes analyses suggest potential utility of the therapy on functional as well as clinical outcomes. Finally, our analyses of therapeutic mechanisms suggest changes in the treatment target — emotional imagery — were associated with symptom change (Supplementary Materials).

We note that the main aim of this paper is not to make an argument about treatment efficacy, but rather to demonstrate the mathematical approach used. Within this longitudinal framework, the single case series method provides the best design to demonstrate the applicability of this methodological innovation at the level most relevant to clinical care—that of the individual patient. An appropriate control is provided by the time-series before treatment. Limitations of the study include that this was a case series with a small sample size, and no parallel control condition. However, the absence of spontaneous improvement during the pre-treatment baseline (Supplementary Materials) indicates that symptom reduction over treatment was unlikely to be simply owing to the passage of time. Unlike in other psychiatric disorders, mood trajectories are not expected to improve spontaneously in bipolar disorder^60^ as instability is persistent and mood constantly fluctuates.^19, 61, 62^ Another limitation is that perhaps daily mood monitoring itself promoted well-being, and this could be tested in future research. The sample is not representative of the wider population, comprising mainly females who were Caucasian, studying or in work, thus caution must be drawn in generalizing from results. However, overall, case series designs are useful at the early stages of treatment innovation where a larger clinical trial is not yet warranted and a detailed individual patient picture useful.^63^ Future studies could include comparative-efficacy and cost-effectiveness testing. Finally, whether improvements in mood instability measured over 4 weeks of daily monitoring are predictive of longer term outcomes beyond the 24-week follow-up, remains to be tested.

We believe that a time-series analysis of daily mood scores collected in everyday life could provide a template for exploring individualized behavioural markers of treatment response. Subsyndromal mood instability is a critical outcome for bipolar disorder in its own right. New measurement approaches^64, 65^ could be applied to any type of bipolar treatment whether pharmacological or psychological. As the quest for biomarkers in bipolar disorder remains problematic, our proposed method of using easy-to-collect computerized/mobile phone self-report data with the aid of probability mathematical models is a pragmatic step. It is noninvasive and inexpensive compared with developing alternatives using neuroimaging or blood sampling for inflammatory markers. It allows development of bespoke time-series that capture clinically meaningful mood instability patterns at the individual level. Future research might seek to consider other disorders characterized by mood instability (for example, borderline personality disorder), or other mood monitoring time schedules ideally based on individual patient need. Extension to preventative mental health, given that children and young people with subsyndromal mood instability have a higher risk for developing bipolar disorder,^18, 61^ will also be crucial. Critically, however, compared with existing methods that require months (via weekly mood monitoring) or even years (via relapse or hospitalization rates) before the efficacy of a treatment can be evaluated, the current approach requires only 1 month of data to assess mood instability. This may provide a useful tool to accelerate the much-needed treatment innovation for bipolar disorder.

## Figures and Tables

**Table 1:** Baseline characteristics of the study cohort (*N*=14) including demographic details, bipolar diagnosis, comorbidities and illness variables, and medication

*Category*	n *(%)/mean (s.d.)*
*Demographic characteristics*	
Age at study intake, years, mean (s.d.)	37.00 (11.82)
Gender, *n* (%)	
Female	12 (86)
Male	2 (14)
Ethnicity, *n* (%)	
White British	11 (79)
White other	3 (21)
	
*Clinical characteristics*	
Bipolar disorder, *n* (%)	
Type 1	9 (64)
Type 2	5 (36)
DSM-5 anxiety specifier, *n* (%)	
Mild	4 (29)
Moderate	4 (29)
Moderate–severe	6 (43)
Comorbidity and clinical course, *n* (%)	
History of psychosis	3 (21)
Current depressive episode	7 (50)
Current comorbid anxiety disorder	9 (64)
Past comorbid anxiety disorder	3 (21)
History of other Axis I disorders	5 (36)
Bipolar illness variables, mean (s.d.)	
Age at illness onset, years	21.07 (10.48); range: 7–48
Number of depressive episodes (past 6 months)	1.29 (0.83); range: 0–3
Duration of depressive episodes (past 6 months) in weeks	11.67 (6.39); range: 5–20
Number of (hypo)manic episodes (past 6 months)	0.79 (0.89); range: 0–3
Duration of (hypo)manic episodes (past 6 months) in weeks	3.13 (2.03); range 1–6
Number of suicide attempts (lifetime)	0.86 (1.46); range: 0–5
Number of hospitalizations (lifetime)	0.93 (2.37); range: 0–7
Number of depressive episodes (lifetime), *n* (%)	
0–4 episodes	4 (29)
5–9 episodes	2 (14)
>10 episodes	8 (57)
Medication at screening, *n* (%)	
Lithium	6 (43)
Anticonvulsants	5 (36)
Antipsychotics	5 (36)
Antidepressants	3 (21)
None	1 (7)

**Table 2:** Weekly depression (QIDS-SR) and anxiety (BAI) scores for the 14 participants combined, aggregated over the pre-treatment baseline (4/5/6 weeks) and over the post-treatment (4 weeks) period

*Outcome measure*	*Pre-treatment (mean±s.d.)*	*Post-treatment (mean±s.d.)*	t*(df),* P*-value*	*Cohen's* d
Weekly QIDS-SR	8.94±3.55	4.41±2.87	*t*(13)=3.86, *P*=0.002	*d*=1.40
Weekly BAI	13.71±4.37	4.80±5.02	*t*(13)=6.55, *P*<0.001	*d*=1.89

Abbreviations: BAI, Beck Anxiety Inventory; QIDS-SR, Quick Inventory of Depressive Symptomatology Self-Report.

**Table 3:** Daily mood scores (QIDS-SR and anxiety ratings) for all 14 participants combined, aggregated over 28 days pre-treatment and 28 days post-treatment

*Outcome measure*	*Pre-treatment (mean±s.d.)*	*Post-treatment (mean±s.d.)*	t*(df),* P*-value*	*Cohen's* d
Daily QIDS-SR	7.19±3.55	3.79±2.59	*t*(13)=2.99, *P*=0.010	*d*=1.09
Daily anxiety	1.77±0.62	0.87±0.82	*t*(13)=4.64, *P*<0.001	*d*=1.24

Abbreviation: QIDS-SR, Quick Inventory of Depressive Symptomatology Self-Report.

## Supplementary Material

Supplementary Informations
