# Supplementary material for: Applications of time-series analysis to mood fluctuations in bipolar disorder to promote treatment innovation: a case series
Source: Transl Psychiatry. 2016 Jan 26;6(1):e720–. doi: 10.1038/tp.2015.207 (PMC5068881; doi:10.1038/tp.2015.207)
Supplement: Supplementary Informations [file tp2015207x1.docx]

List of Supplementary Materials and Methods

- - Participant recruitment and screening
  - Computerized monitoring system security
- **Measures**
  - Structured Clinical Interview for DSM-IV Disorders (SCID)
  - Ratings of imagery treatment target
  - Mania Symptoms
- **Additional clinical and functioning measures**
  - Affective Lability Scale – short version (ALS-18)
  - Longitudinal Interval Follow up Evaluation – Range of Impaired Functioning Tool scale (LIFE-RIFT)
  - Beck Hopelessness Scale (BHS)
  - QIDS Suicidality Rating
  - Medication Compliance Questionnaire
- **Statistical analysis**
- **Time-Series Analysis**
  - Probability Distributions and Likelihoods
  - Changes in ratings of psychological treatment target (imagery) and associated changes in mood over treatment (treatment mechanism)
  - Additional outcome measures over 24 weeks follow-up
- **Supplementary Results**
  - Weekly mood monitoring scores remain consistent over the pre-treatment baseline periods
  - Changes in ratings of the imagery treatment target show some association with change in symptoms
- **Time-Series Model Results**
  - Treatment intervention affects temporal structure of daily mood profiles: aggregated patient analysis
  - Treatment intervention affects individual daily mood instability profile
- **Additional outcome measures over 24 weeks follow-up**
  - Weekly Depression and Anxiety scores
  - Clinical relapses 24 weeks pre- and post-treatment
  - Clinical and functioning outcomes assessed at pre-treatment, post-treatment, 4, 12, and 24 weeks follow-up
  - Intervention effects on self-reported (hypo)manic symptoms

Supplementary figures

- **Fig. S1.**Weekly scores for Depression (QIDS; dashed line) and Anxiety (BAI; solid line) for each individual case pre-treatment (4/5/6 week baseline), post-treatment, and 24 weeks follow-up. Participants presented in order of starting mood monitoring.
- **Fig. S.2.** Fit of the overall observed probabilities of participant QIDS scores pre- and post-treatment (pink dots) to a Gamma probability model (solid black line)

Supplementary Tables

- **Table S1.** Additional baseline characteristics of the study cohort (*N*=14) including demographic characteristics, specification of current and past co-morbid anxiety and other Axis I disorders.
- **Table S.2.** AIC and AIC weights (wAIC) for autoregressive models fitted across all participants (pre- and post-treatment). Figures in bold show best fitting models for each group (pre- or post-treatment).
- **Table S.3.** QIDS score summary statistics (mean, standard deviation (SD) and coefficient of variation (CV)) for each participant (in order of starting mood monitoring) pre- and post-treatment.
- **Table S.4.** Scores for Affective Lability, impairments in functioning, hopelessness and suicidality, and Medication Compliance across the face-to-face pre-treatment, post-treatment and follow up assessments.

**Supplementary Materials and Methods**

**Participant recruitment and screening**

Inclusion criteria were as follows: (a) ages 18 – 65, (b) adequate English for completion of the outcome measures, (c) *Diagnostic and Statistical Manual of Mental Disorders*, fourth edition (DSM-IV) diagnosis of Bipolar Disorder (I or II or NOS), as indicated by the Structured Clinical Interview for DSM-IV [SCID:^1^](#_ENREF_1) (d) willing to complete the required mood monitoring throughout the duration of the study, (e) successful completion of pre-treatment baseline monitoring (at least 23 out of 28 daily mood measures in the 4-week active run-in phase of the study), (f) willing to have regular treatment reports and letters sent to relevant health professionals, (g) can commit to attending 10 consecutive weekly sessions, including questionnaire completion, and follow up period as required by the study, (h) resident within the geographical areas covered by the U.K. National Health Service (NHS) mental health service in which the treatment is delivered.

Exclusion criteria were (a) learning difficulties, organic brain disease, severe neurological impairment, (b) current severe substance or alcohol misuse, (c) current manic episode according to DSM-IV criteria, indicated by the SCID, (d) current active psychotic symptoms, (e) presence of active suicidal risk (a score of 2 or more on item 12 of the QIDS[^2^](#_ENREF_2) confirmed by clinical opinion), (f) unwilling to engage actively in treatment, (g) taking part in concurrent treatment studies for BD. Additionally, given the treatment target was current anxiety/mood, participants without clinical levels of symptoms were not eligible for treatment, i.e. where the baseline period indicated no significant levels of anxiety (at least one BAI score in the “moderate” range or higher, i.e. ≥ 16)[^3^](#_ENREF_3) or depression (at least two QIDS scores in the “moderate” range or higher, i.e. ≥10).[^2^](#_ENREF_2)

Reasons for exclusion were: presence of active suicidal risk (*n* = 1); moving out of area (*n* = 3), unable to attend sessions due to housing eviction (*n* = 1); lacked clinical levels of target symptoms as defined above (*n* = 6); did not attend therapy assessment (*n* = 1). Two additional participants were eligible but either chose not to take part in the study (*n* = 1) or did not respond to further contact (*n* = 1). None were excluded due to inability to complete daily baseline monitoring.

**Table S1.** Additional baseline characteristics of the study cohort (*N*=14) including demographic characteristics, specification of current and past co-morbid anxiety and other Axis I disorders.

| **Characteristic** | ***n* (%) / mean (SD)** |
| --- | --- |
| **Demographic characteristics** |  |
| Years of Education, mean (SD) | 15.36 (2.48) |
| Employment status, *n* (%) |  |
| Employed (paid or unpaid) | 10 (71) |
| Student | 3 (21) |
| Homemaker | 1 (7) |
| Unemployed | 0 |
| Marital Status, *n* (%) |  |
| Single | 9 (64) |
| Married or cohabiting | 3 (21) |
| Separated or divorced | 1 (7) |
| **Clinical characteristics** |  |
| Current Comorbid Anxiety Disorder, *n* (%) |  |
| Social anxiety | 6 (43) |
| Generalized anxiety disorder | 2 (14) |
| Specific Phobia | 4 (29) |
| Agoraphobia without panic disorder | 1 (7) |
| Past Comorbid Anxiety Disorder, *n* (%) |  |
| Social anxiety | 3 (21) |
| Generalized anxiety disorder | 1 (7) |
| Panic Disorder with Agoraphobia | 1 (7) |
| History of other Axis I Disorder, *n* (%) |  |
| Alcohol dependence | 1 (7) |
| Substance abuse | 1 (7) |
| Substance dependence | 1 (7) |
| Bulimia | 1 (7) |
| Anorexia | 2 (14) |

**Computerized monitoring system security**

The two servers supporting the web-based monitoring system were protected by a firewall that restricted access to network ports. Access to the website for completing the questionnaires was only via an https protocol to ensure that participants’ responses were encrypted in transit. The web server hosting the website stored no personally identifiable information, and participants logged in using an anonymous user id and password. Participants’ personal data required for sending the automated reminders (name, email address, mobile phone number) were stored on the separate database server, which could not be accessed by computers external to the host institution’s network.

**Measures**

**Structured Clinical Interview for DSM-IV Disorders (SCID)**

Number and duration of depressive and hypomanic or manic relapses over 24 weeks before and after treatment were computed using DSM-IV criteria for depressive and manic/hypomanic episodes, assessed retrospectively via the SCID.[^1^](#_ENREF_1) Pre-treatment relapses were assessed at screening covering the previous 24 weeks. Post-treatment relapses were computed adding up the relapses assessed at 4, 12 and 24 weeks follow-ups each covering time elapsed from the previous assessment (total = 24 weeks). Once diagnostic criteria for a past depressive, manic/hypomanic episode over the previous 24 weeks or since the last assessment was established, participants were asked to estimate the *duration* of the episodes (number of weeks).

Number of anxiety disorders was computed using DSM-IV criteria for current anxiety disorders assessed retrospectively via the SCID conducted at screening, 4, 12 and 24 weeks follow up assessments.

**Ratings of imagery treatment target**

Mental imagery treatment targets were assessed using the Mental Imagery in Bipolar Disorder Questionnaire (MICQ-BD) at the face-to-face interviews pre- and post-treatment. This is a 14 item self-report bespoke instrument developed as part of our pilot psychology service for bipolar disorder. It was designed to assess patient responses to mental imagery, for example, question 5 reads ‘*When an unhelpful mental image popped up, I could disengage from it*’. Ratings were on a 5-point scale from “not at all” to “a lot”. In the current study, the internal reliability coefficient alpha of the MICQ-BD at the pre-treatment assessment was satisfactory (α = 0.70).

Additionally, two imagery ratings were collected weekly via the same computerised system used for weekly mood monitoring, both pre- and post-treatment e.g. “*To which extent could you understand the role that the image(s) play in changing your mood?*”. Ratings were from 1 (‘*not at all’*) to 10 (‘*extremely’*).

**Mania symptoms**

Mania-related symptoms over the past 7 days were assessed using the Altman Self-Rating Scale for Mania [ASRM^4^](#_ENREF_4). The ASRM is a 5-item self-report questionnaire covering ‘positive mood’, ‘self-confidence’, ‘sleep patterns’, ‘speech and ‘activity levels’. Ratings are made on a five-point scale (0-4) with a maximum score of 20. The ASRM is an established psychometric tool for detecting mania among BD patients with a good internal consistency (α = 0.79).[^4^](#_ENREF_4), [^5^](#_ENREF_5)

Note that weekly mania symptoms were collected for clinical purposes (monitoring during therapy) but are not a pre-specified outcome measure and are not included in the analysis.

**Additional clinical and functioning measures**

**Affective Lability Scale – short version (ALS-18)**

The Affective Lability Scale Short Version ([ALS-18)^6^](#_ENREF_6) is an 18 items self-report scale measuring trait lability in affect during euthymic and other affective states including depression, anxiety, elation and anger. Ratings are made on a 4-point scale with a maximum score of 72: ‘A = *very characteristic of me, extremely descriptive’ (4 points), ‘*B = *rather characteristic of me, quite descriptive’ (3 points), ‘*C = *rather uncharacteristic of me, quite undescriptive’ (2 points),‘* D = *very uncharacteristic of me, extremely undescriptive (1 point)’.* For example, Question 3 reads ‘one moment I am feeling OK and then the next minute I’m tense, jittery and nervous’.

**Longitudinal Interval Follow up Evaluation – Range of Impaired Functioning Tool scale (LIFE-RIFT)**

The Longitudinal Interval Follow up Evaluation – Range of Impaired Functioning Tool (LIFE-RIFT)[^7^](#_ENREF_7) is a brief scale for people with affective disorders. Four functional areas are assessed: Employment, Interpersonal Relations, Satisfaction, and Recreation. Ratings are made on a 1 to 5 scale in each area, with lower ratings indicating higher functioning. A total score is also generated. Descriptions for each rating are given to aid judgement, for example, in the Work section: 1 = ‘*no impairment – high level. Worked as much as someone in her/his social situation would be expected to work, and worked at a high level.*’

**Beck Hopelessness Scale (BHS)**

The Beck Hopelessness Scale[^8^](#_ENREF_8) is a 20-item self-report instrument with true (=1 point) /false (=0 points) statements with a maximum score of 20. It assesses 3 aspects of hopelessness: feelings about the future, motivation and expectations. For example: ‘*Things just won’t work out the way I want them to*’ and ‘*I can look forward to more good times than bad times*’. The BHS demonstrates good internal consistency (alpha = 0.93)[^9^](#_ENREF_9) and has high reliability in psychiatric samples.[^10^](#_ENREF_10) High levels of hopelessness on the BHS has been linked to suicidal ideations in bipolar patients.[^11^](#_ENREF_11), [^12^](#_ENREF_12)

**QIDS Suicidality Rating**

Scores on the suicide item from the Quick Inventory of Depressive Symptomatology Self-Report QIDS-SR[^2^](#_ENREF_2) were used to assess changes in suicidality from pre to post-treatment. The item asks participants to rate their thoughts of death or suicide over the last seven days where 0= *‘I do not think of suicide or death’*, 1= *‘I feel that life is empty of wonder if it is worth living’*, 2= *‘I think of suicide or death several times over the past 7 days for several minutes’*, 3= *‘I think of suicide or death several times a day in some detail or I have made specific plans for suicide or have actually tried to take my life’*.

**Medication Compliance Questionnaire**

The Medication Compliance Questionnaire[^13^](#_ENREF_13) is a one-item scale asking participants to report whether over the past six months or since their last face-to-face assessment they: “*never missed taking medication*” (=0 points), “*missed taking medication once or twice*”, “*missed taking medication between 3 and 7 times*”, “*missed taking medication more than 7 times*” or “*stopped taking medication altogether*”.[^13^](#_ENREF_13) Scores on the questionnaire range from 0 to 4.

**Statistical analysis**

**Time-Series Analysis**

**Probability Distributions and Likelihoods**

Pre- and post-treatment mood scores are well characterized by a Gamma distribution (Figure S2). This is also well supported by our previous analyses [e.g.,^14^](#_ENREF_14) and we use this probability distribution to derive appropriate likelihood structures for the time-series analysis. This probability distribution function (PDF) is characterized by two parameters: a rate (*r*) and a shape (*s*) parameter:

$$f(x;r,s)=\frac{x^{(r-1)}s^{r}\exp(-sx)}{\Gamma(r)}$$

where *x* is an observation and $\Gamma(r)$ is a gamma function ([r-1]!). The mean from this distribution is *μ*=*r/*s and the variance is *σ^2^*=*r*/*s*^2^. From a time-series of *T* mood scores we use this Gamma probability distribution function to construct an appropriate likelihood (L(**P**|**Y**)) where for an AR(1) (or TAR(1)) model the first point in the time-series is conditional on the mean and subsequent points on the previous observation:

$$L(\mathbf{P}|\mathbf{Y})=\frac{Y_{1,j}^{r-1}\left(\frac{r}{\mu_{1}}\right)^{r}\exp\left(-\left(\frac{r}{\mu_{1}}\right)Y_{1,j}\right)}{\Gamma(r)}\prod_{k=2}^{T} \frac{Y_{k,j}^{r-1}\left(\frac{r}{\mu_{k}}\right)^{r}\exp\left(-\left(\frac{r}{\mu_{k}}\right)Y_{k,j}\right)}{\Gamma(r)}$$

where **P** is an unknown parameter set associated with the time-series model fitted to the mood observations (e.g. AR(1)), **Y** is a set of mood score observations, *μ_1_* is the mean of the *j^th^* participant’s mood series and *μ*_j_ is then the expected mood score (derived from the underlying time-series model e.g., AR(1)) for the *k*^th^ time point. *Y_k,j_* is then the *k*^th^ mood score (out of T) for the *j^th^* participant. Similar extensions to the likelihood are made to fit AR(2) or TAR(2) models.

To determine maximum likelihood parameter estimates for the different time-series models (AR(1), AR(2), TAR(1), TAR(2)), we minimize the negative log-likelihood using an expectation-maximization method to deal with missing values within a modified simplex algorithm.[^14^](#_ENREF_14) Goodness of fit between model predictions and mood observations is evaluated through the use of AIC scores[^15^](#_ENREF_15) and one-step ahead predictions.

**Changes in ratings of psychological treatment target (imagery) and associated changes in mood over treatment (treatment mechanism)**

~~To~~ We tested if improvements in ratings of imagery treatment targets are associated with concurrent improvements in depression and anxiety. C~~c~~hange scores of the Mental Imagery in Bipolar Disorder Questionnaire (MICQ-BD) ~~and~~ as well as the imagery treatment target ratings measured weekly in everyday life, were each correlated with change scores of weekly QIDS (depression) and BAI (anxiety) total scores from aggregated baseline to aggregated post-treatment assessments. ~~were computed.~~

**Additional outcome measures over 24 weeks follow-up**

One-way repeated measures analysis of variance was used to assess differences in number of relapses, relapse duration, number of comorbid anxiety disorders and average scores on the additional clinical and functioning measures over time with post-hoc tests carried out using paired t-tests with a Bonferroni correction. T-tests and analysis of variance were used to compare groups with non-parametric Wilcoxon and Friedman tests used if residuals outputted from parametric tests were non-normally distributed[^16^](#_ENREF_16) or if responses showed a limited range of values (e.g. only taking values, 0, 1 and 2) which motivated considering these measures as ordered ranks. All tests were two-tailed.

**Supplementary Results**

**Weekly mood monitoring scores remain consistent over the pre-treatment baseline periods**

Linear regression estimates of individual weekly anxiety and depression scores during baseline indicated no systematic trend towards symptom decrease during baseline: For 11 participants there was no change, and for 3 participants symptoms changed: Participant 5 (randomized to a 4 week baseline) showed an increase in anxiety ratings (β = 0.99, *p* = 0.006); participant 10 (randomized to a 5-week baseline), showed an increase in depression ratings (β = 0.95, *p* = 0.014); and participant 14 (randomized to a 6-week baseline) showed a decrease in anxiety ratings (β = -0.92, *p* = 0.009) during baseline.

**Changes in ratings of the imagery treatment target show some association with change in symptoms**

Ratings on the imagery treatment target questionnaire (MICQ-BD –at face-to-face interviews) improved from pre-treatment (M = 35.14, SD = 8.24) to post-treatment (M = 54.54, SD = 7.81), *t*(12) = 5.40, *p* < 0.001 , *d* = 2.42. The correlation between improvements on these ratings with improvements in weekly symptoms were for depression (BDI), *r* = -0.54, *p* = 0.058 and anxiety (BAI) *r* = -0.76, *p* = 0.003. Ratings of the imagery treatment target measured weekly in everyday life (via the computerized system) improved from pre-treatment (M = 3.87, SD = 2.38) to post-treatment (M = 7.07, SD = 1.70), *t*(13) = 3.20, *p* = 0.007, *d* = 1.55. The equivalent correlations were for depression, *r* = -0.63, *p* = 0.016 and anxiety *r* = -0.32, *p* = 0.266.

**Time-Series Model Results**

**Treatment intervention affects temporal structure of daily mood profiles: aggregated patient analysis**

AIC values (and AIC weights) for AR(1), AR(2), TAR(1) and TAR(2) time-series models fitted across all participant time-series pre- and post- treatment are shown in **Table S.2.**

**Table S.2**. AIC and AIC weights (wAIC) for autoregressive models fitted across all participants (pre- and post-treatment). Figures in bold show best fitting models for each group (pre or post-treatment).

| **Autoregressive structure** | **Pre-Treatment** | | **Post-Treatment** | |
| --- | --- | --- | --- | --- |
|  | **AIC** | **wAIC** | **AIC** | **wAIC** |
| AR(1) | 1152.644 | <0.0001 | 606.681 | <0.0001 |
| AR(2) | 1099.011 | 0.0269 | **570.071** | **0.999** |
| TAR(1) | 1151.067 | <0.0001 | 609.012 | <0.0001 |
| TAR(2) | **1091.836** | **0.973** | 1086.203 | <0.0001 |

The best fitting model for the pre-treatment pooled participant time-series was a threshold autoregressive model of order 2:

where $\bar{Y}$is the mean of the time-series. The parameter estimates (with the 95% C.I.) for this time-series model were *a_0_*=1.198±0.3, *a_1_*=0.479±0.074, and *a_2_*=0.422±0.141, *a_3_*=1.031±0.135, *a_4_*=0.268±0.043, and *a_5_*=0.383±0.073.

The best fitting model (with 95% CI) for the post-treatment pooled participant time-series was an autoregressive model of order 2:

$$Y_{t}=a_{0}+a_{1}Y_{t-1}+a_{2}Y_{t-2}$$

The parameter estimates (with the 95% C.I.) for this time-series model were *a_0_*=0.565±0.265, *a_1_*=0.410±0.04, and *a_2_*=0.322±0.225.

In-sample (one-step ahead) predictions based on these models were generated for each participant and the goodness of fit for these models are illustrated in Figure 2 (see main text).

**Treatment intervention affects individual daily mood instability profile**

**Table S.3** QIDS score summary statistics (mean, standard deviation (SD) and coefficient of variation (CV)) for each participant (in order of starting mood monitoring) pre- and post-treatment.

| **Participant** | **Pre-Treatment Summary Statistics** | | | **Post-Treatment Summary Statistics** | | |
| --- | --- | --- | --- | --- | --- | --- |
|  | **Mean** | **SD** | **CV** | **Mean** | **SD** | **CV** |
| P1 | 9.11 | 3.81 | 0.418 | 1.86 | 1.04 | 0.562 |
| P2 | 3.74 | 1.83 | 0.490 | 4.78 | 1.76 | 0.367 |
| P3 | 6.20 | 4.74 | 0.765 | 1.09 | 2.00 | 1.830 |
| P4 | 13.93 | 2.93 | 0.210 | 5.18 | 1.25 | 0.241 |
| P5 | 8.08 | 3.47 | 0.430 | 2.52 | 1.40 | 0.555 |
| P6 | 3.36 | 2.70 | 0.803 | 1.00 | 1.70 | 1.700 |
| P7 | 4.43 | 2.23 | 0.505 | 3.88 | 1.45 | 0.374 |
| P8 | 6.56 | 4.27 | 0.652 | 3.70 | 3.30 | 0.892 |
| P9 | 6.12 | 4.06 | 0.664 | 3.00 | 3.70 | 1.233 |
| P10 | 11.41 | 2.45 | 0.215 | 5.54 | 0.64 | 0.115 |
| P11 | 2.57 | 2.35 | 0.917 | 10.36 | 2.48 | 0.240 |
| P12 | 8.37 | 5.96 | 0.712 | 1.14 | 1.30 | 1.135 |
| P13 | 12.54 | 5.81 | 0.464 | 6.64 | 3.67 | 0.553 |
| P14 | 4.29 | 2.45 | 0.571 | 2.39 | 1.99 | 0.831 |
|  |  |  |  |  |  |  |
| Overall | 7.25 | 5.03 | 0.694 | 3.83 | 3.32 | 0.866 |

**Additional outcome measures over 24 weeks follow-up**

**Weekly Depression and Anxiety scores**

Weekly scores for Depression (QIDS) and Anxiety (BAI) for each individual case pre-treatment, post-treatment, and 24 weeks follow-up are shown in Figure S.1.

**Clinical relapses 24 weeks pre- and post-treatment**

There was a significant reduction in the number of depressive episodes over 24 weeks following treatment compared to 24 weeks pre-treatment (pre-treatment: Mean = 1.21, SD = 0.7; post-treatment: Mean = 0.43, SD = 0.76; *z* = 2.30, *p* = 0.022, *d* = 1.56), but not in the number of (hypo) manic episodes (pre-treatment: Mean = 0.79, SD = 0.89; post-treatment: Mean = 0.36, SD =0 .84; *z* = 1.29, *p* = 0.199, *d* = 0.73). Overall, the amount of time spent in depression during 24 weeks reduced from 10.0, SD = 7.24 weeks pre-treatment to 1.32, SD = 2.52 weeks post-treatment (*z* = 3.07, *p* = 0.002, *d* = 2.87). The amount of time spent in (hypo)mania during 24 weeks did not significantly reduce from pre-treatment to post-treatment (pre-treatment: Mean = 1.71, SD = 2.20; post-treatment: Mean = 0.40, SD = 0.86; *z* = 1.69, *p* = .090, *d* = 1.01).

**Clinical and functioning outcomes assessed at pre-treatment, post-treatment, 4, 12, and 24 weeks follow-up**

Number of comorbid anxiety disorders and scores on Affective Lability (ALS-18), impairments in functioning (LIFE-RIFT), hopelessness (BHS) and suicidality (QIDS Suicidality Rating), and Medication Compliance are summarized in Table S.4.

**Intervention effects on self-reported (hypo)manic symptoms**

While mania was not a pre-specified primary or secondary outcome measure, it is noted that there was no significant improvement in mean self-reported weekly ratings of manic symptoms from pre-treatment (M = 2.98, SD = 2.95) to post-treatment (M = 2.23, SD = 2.26), *t*(13) = 0.74; *p* = 0.471; *d* = 0.29.

**Table S.4.** Scores for affective lability, impairment in functioning, hopelessness, suicidality, and medication compliance across the face-to-face pre-treatment, post-treatment and the follow up assessments.

| **Outcome measure** | **Pre-treatment**  **(mean ± SD)** | **End of-treatment**  **(mean ± SD)** | **4 week FU**  **(mean ± SD)** | **12 week FU**  **(mean ± SD)** | **24 week FU**  **(mean ± SD)** | **Time** | **Pairwise difference (if overall main effect of time) Bonferroni corrected pairwise t-tests** |
| --- | --- | --- | --- | --- | --- | --- | --- |
| Anxiety comorbidity (SCID) | 1.14 ± 1.10 | N/A* | 0.36 ± 0.50 | 0.29 ± 0.61 | 0.64 ± 1.01 | *F*(3,39) = 4.97, p = 0.005 | No pairwise comparisons have *p* < 0.05 |
| ALS-18 | 46.36 ± 11.26 | 35.54 ± 10.49 | 35.43 ± 10.92 | 36.36 ± 12.58 | 34.71 ± 12.74 | *F*(4,48) = 6.75 , *p* < 0.001 | Pre vs 4w *t*(12) = 3.77, *p* = 0.027, *d* = 0.99; Pre vs 12w *t*(12) = 3.58, *p* = 0.038, *d* = 0.84; Pre vs 24w *t*(12) = 4.62, *p* = 0.006, *d* = 0.97 |
| LIFE-RIFT | 10.57 ± 3.08 | 7.67 ± 3.08 | 7.29 ± 1.94 | 8.08 ± 2.64 | 7.15 ± 3.26 | *F*(4,36) = 3.34, *p* = 0.020 | Pre vs 4w *t*(9) = 3.87, *p* = 0.038, *d* = 1.27 |
| BHS | 9.21 ±3.17 | 3.15 ± 2.51 | 4.43 ± 4.72 | 3.43 ± 2.38 | 2.57 ± 2.31 | *F*(4,48) = 19.41 , *p* < 0.001 | Pre vs Post *t*(12) = 6.11, *p* = 0.001, *d* = 2.11; Pre vs 4w *t*(12) = 4.72, *p* = 0.005, *d* = 1.17; Pre vs 12w *t*(12) = 5.66, *p* = 0.001, *d* = 2.06; Pre vs 24w *t*(12) = 6.03, *p* =0.001, *d* = 2.39 |
| Suicidality Rating | 0.36 ± 0.63 | 0.23 ± 0.60 | 0.00 ± 0.00 | 0.00 ± 0.00 | 0.00 ± 0.00 | *χ^2^*  (4) = 8.00, *p* = 0.092 |  |
| Medication Compliance | 1.50 ± 0.67 | 2.00 ± 1.41 | 2.09 ± 1.70 | 1.60 ± 0.97 | 1.91 ± 0.94 | *χ^2^* (4) = 3.33, *p* = 0.504 |  |

Anxiety comorbidity (SCID) = number of anxiety disorders assessed by the Structured Clinical Interview for DSM-IV Disorders; ALS-18 = Affective Lability Scale – short version; BHS = Beck Hopelessness Scale; LIFE-RIFT = Longitudinal Interval Follow up Evaluation - Range of Impaired Functioning; QIDS = Quick Inventory of Depressive Symptomatology; Suicidality Rating = assessed with item 12 on ‘thoughts of death or suicide’ of the QIDS. *SCID was only administered at 4, 12 and 24 weeks FU

**Fig. S1.**Weekly scores for Depression (QIDS; dashed line) and Anxiety (BAI; solid line) for each individual case pre-treatment (4/5/6 week baseline), post-treatment, and 24 weeks follow-up. Participants presented in order of starting mood monitoring.


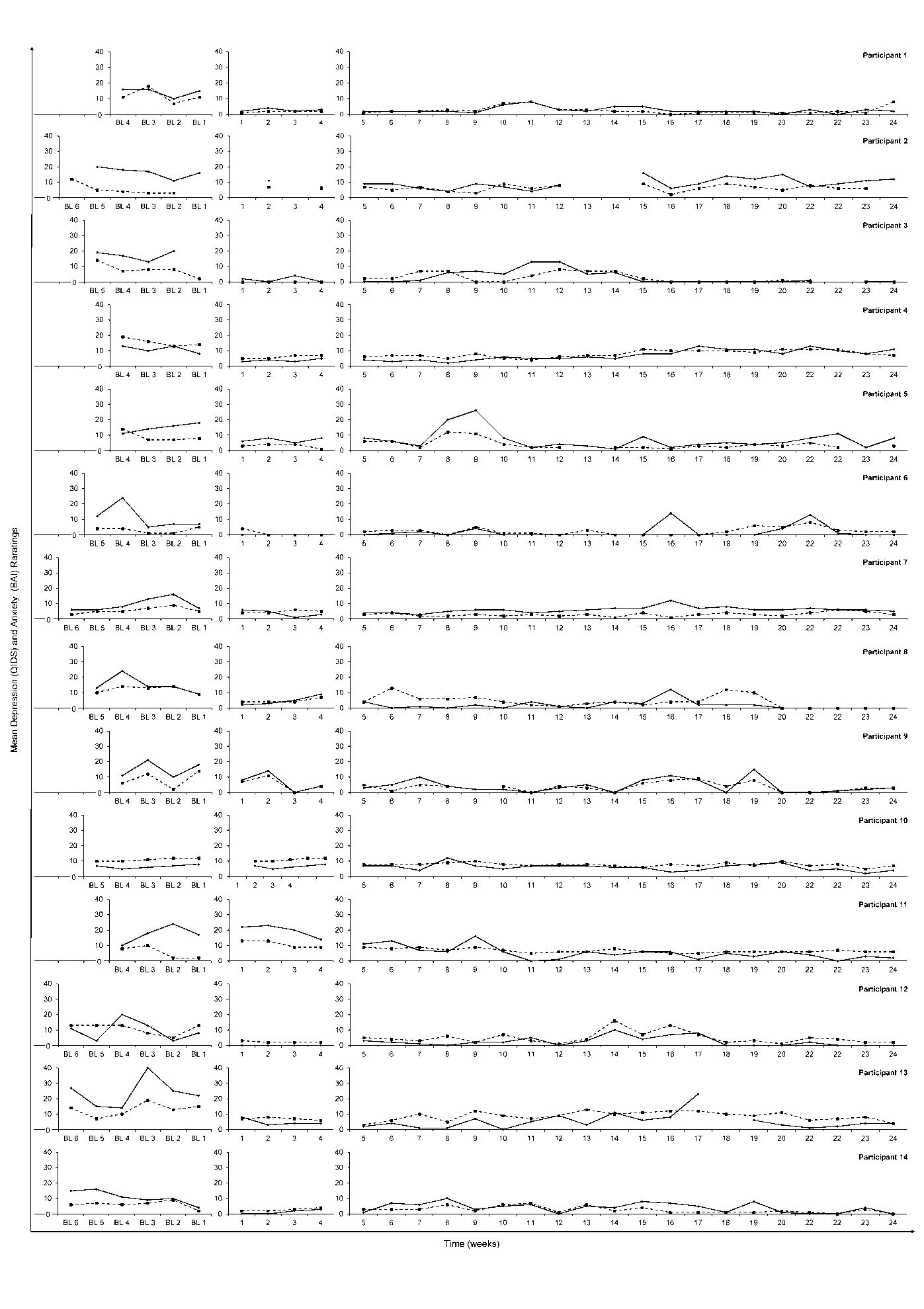


**Fig. S.2.** Fit of the overall observed probabilities of participant QIDS scores pre- and post-treatment (pink dots) to a Gamma probability model (solid black line).

**References for supplementary materials**

1. First MB, Spitzer RL, Gibbon M, Williams JBW. *Structured Clinical Interview for DSM-IV-TR Axis I Disorders, Research Version, Patient Edition. (SCID-I/P)*. Biometrics Research, New York State Psychiatric Institute: New York, 2002.

2. Rush JA, Trivedi MH, Ibrahim HM, Carmody TJ, Arnow B, Klein DN*, et al*. The 16-item quick inventory of depressive symptomatology (QIDS), clinician rating (QIDS-C), and self-report (QIDS-SR): A psychometric evaluation in patients with chronic major depression. *Biological Psychiatry* 2003; **54**(5)**:** 573-583.

3. Beck AT, Steer RA. *Beck Anxiety Inventory Manual*. Psychological Corporation: San Antonio, 1993.

4. Altman EG, Hedeker D, Peterson JL, Davis JM. The Altman self-rating mania scale. *Biological Psychiatry* 1997; **42**(10)**:** 948-955.

5. Altman EG, Hedeker D, Peterson JL, Davis JM. A comparative evaluation of three self-rating scales for acute mania. *Biological Psychiatry* 2001; **50**(6)**:** 468-471.

6. Oliver MNI, Simons JS. The affective lability scales: Development of a short-form measure. *Personality and Individual Differences* 2004; **37**(6)**:** 1279-1288.

7. Leon AC, Solomon DA, Mueller TI, Endicott J, Posternak M, Judd LL*, et al*. A brief assessment of psychosocial functioning of subjects with bipolar I disorder: the LIFE-RIFT. Longitudinal Interval Follow-up Evaluation-Range Impaired Functioning Tool. *The Journal of nervous and mental disease* 2000; **188**(12)**:** 805-812.

8. Beck AT, Steer RA. *Manual for the Beck Hopelessness Scale*. Psychological Corporation: San Antonio, TX, 1988.

9. Beck AT, Weissman A, Lester D, Trexler L. The measurement of pessimism: the hopelessness scale. *Journal of Consulting and Clinical Psychology* 1974; **42**(6)**:** 861-865.

10. Beck AT, Brown GK, Berchick RJ, Stewart BL, Steer RA. Relationship between hopelessness and ultimate suicide: a replication with psychiatric outpatients. *American Journal of Psychiatry* 1990; **147**(2)**:** 190-195.

11. Umamaheswari V, Avasthi A, Grover S. Risk factors for suicidal ideations in patients with bipolar disorder. *Bipolar disorders* 2014; **16**(6)**:** 642-651.

12. Acosta FJ, Vega D, Torralba L, Navarro S, Ramallo-Farina Y, Fiuza D*, et al*. Hopelessness and suicidal risk in bipolar disorder. A study in clinically nonsyndromal patients. *Comprehensive Psychiatry* 2012; **53**(8)**:** 1103-1109.

13. Lam DH, Watkins ER, Hayward P, Bright J, Wright K, Kerr N*, et al*. A randomized controlled study of cognitive therapy for relapse prevention for bipolar affective disorder: Outcome of the first year. *Archives of General Psychiatry* 2003; **60**(2)**:** 145-152.

14. Bonsall MB, Wallace-Hadrill SMA, Geddes JR, Goodwin GM, Holmes EA. Nonlinear time-series approaches in characterizing mood stability and mood instability in bipolar disorder. *Proceedings of the Royal Society B: Biological Sciences* 2012; **279**(1730)**:** 916-924.

15. Burnham KP, Anderson D. *Model selection and multi-model inference.* . Springer: London, 2002.

16. Andrews DF, Gnanadesikan R, Warner JL. *Methods for assessing multivariate normality. Multivariate Analysis III*. Academic Press: New York, 1973.
